# No-Reflow Phenomenon in Central Retinal Artery Occlusion: Incidence, Risk Factors, and Clinical Implications

**DOI:** 10.1371/journal.pone.0142852

**Published:** 2015-11-23

**Authors:** Seong Joon Ahn, Kyu Hyung Park, Na-Kyung Ryoo, Jeong-Ho Hong, Cheolkyu Jung, Chang-Hwan Yoon, Moon-Ku Han, Se Joon Woo

**Affiliations:** 1 Department of Ophthalmology, Seoul National University College of Medicine, Seoul National University Bundang Hospital, Seongnam, Korea; 2 Department of Neurology, Keimyung University Dongsan Medical Center, Daegu, Korea; 3 Department of Neurology, Seoul National University Bundang Hospital, Seongnam, Korea; 4 Department of Radiology, Seoul National University Bundang Hospital, Seongnam, Korea; 5 Department of Cardiology, Seoul National University Bundang Hospital, Seongnam, Korea; Massachusetts Eye & Ear Infirmary, Harvard Medical School, UNITED STATES

## Abstract

**Purpose:**

To investigate the incidence and risk factors of the no-reflow phenomenon in central retinal artery occlusion (CRAO) patients and to determine its effects on visual and anatomic outcomes.

**Methods:**

In 102 eyes with CRAO in which arterial recanalization was obtained within 1 week from baseline, fluorescein angiography images obtained at baseline and 1 week were retrospectively reviewed. The no-reflow phenomenon in the retina was defined as macular capillary nonperfusion following arterial recanalization on fluorescein angiographs. We investigated the incidence and risk factors for the no-reflow phenomenon and compared the anatomical and visual outcomes between eyes with and without the phenomenon.

**Results:**

Among the 102 CRAO eyes with arterial recanalization, 39 exhibited the no-reflow phenomenon, resulting in an incidence of 38.2%. The incidence among the eyes with treatment-induced and spontaneous recanalization was 43.4% and 15.8%, respectively, and it increased with the CRAO stage. CRAO stage and increased central macular thickness were risk factors for the phenomenon, with an odds ratio of 4.47 [95% confidence interval (CI), 1.19–16.8; P = 0.027] and 1.69 (95% CI, 1.12–2.55; P = 0.012) per 100-μm increase, respectively. The visual outcome was significantly poorer and retinal atrophy and photoreceptor disruption was greater in eyes with the no-reflow phenomenon than in those without.

**Conclusions:**

The no-reflow phenomenon may occur after arterial recanalization in approximately one-third of CRAO patients and can affect anatomical and visual outcomes. This phenomenon may provide an additional explanation regarding the permanent retinal damage and vision loss in eyes with CRAO.

## Introduction

Inadequate tissue perfusion is occasionally observed after successful arterial recanalization, and this event is defined as the no-reflow phenomenon. The concept of no-reflow was first suggested in experimental brain ischemia models wherein the normal flow to brain tissues was not restored after obstruction removal.[1, 2] In clinical settings, myocardial no-reflow, characterized by absence of intramyocardial reperfusion after successful coronary recanalization for myocardial infarction, is well documented.[3, 4] The no-reflow phenomenon has been observed after percutaneous coronary intervention (PCI) in 5% to 50% patients with acute myocardial infarction.[5–8] A strong negative impact of the phenomenon on PCI outcomes, which negates its potential benefits, is clearly reported.[9–13]

Central retinal artery occlusion (CRAO) represents end-organ ischemia of the eye and is analogous to terminal branch occlusion in cerebral stroke and acute myocardial infarction. Four distinct categories exist based on the pathogenesis and clinical characteristics: non-arteritic, non-arteritic with cilioretinal artery sparing, arteritic, and transient non-arteritic.[14] Hayreh and colleagues showed the no-reflow phenomenon in eyes with transient non-arteritic CRAO, where angiography shows normal retinal vascular bed filling with no filling in the macular region.[14] They suggested it as a cause of permanent ganglion cell death and central scotoma.[14, 15] However, the phenomenon itself has received little attention, particularly in terms of incidence, risk factors, and effects on anatomic and visual outcomes.

This study retrospectively reviewed fluorescein angiography (FA) images to investigate the incidence of the no-reflow phenomenon in consecutive patients with non-arteritic CRAO in whom arterial patency was restored either spontaneously or by conservative treatment or intra-arterial thrombolysis (IAT). In addition, the study aimed to determine the risk factors for this phenomenon and evaluate retinal structural changes on optical coherence tomography (OCT) and visual outcomes in eyes with CRAO.

## Methods

### Patient Selection

**Fig 1:**
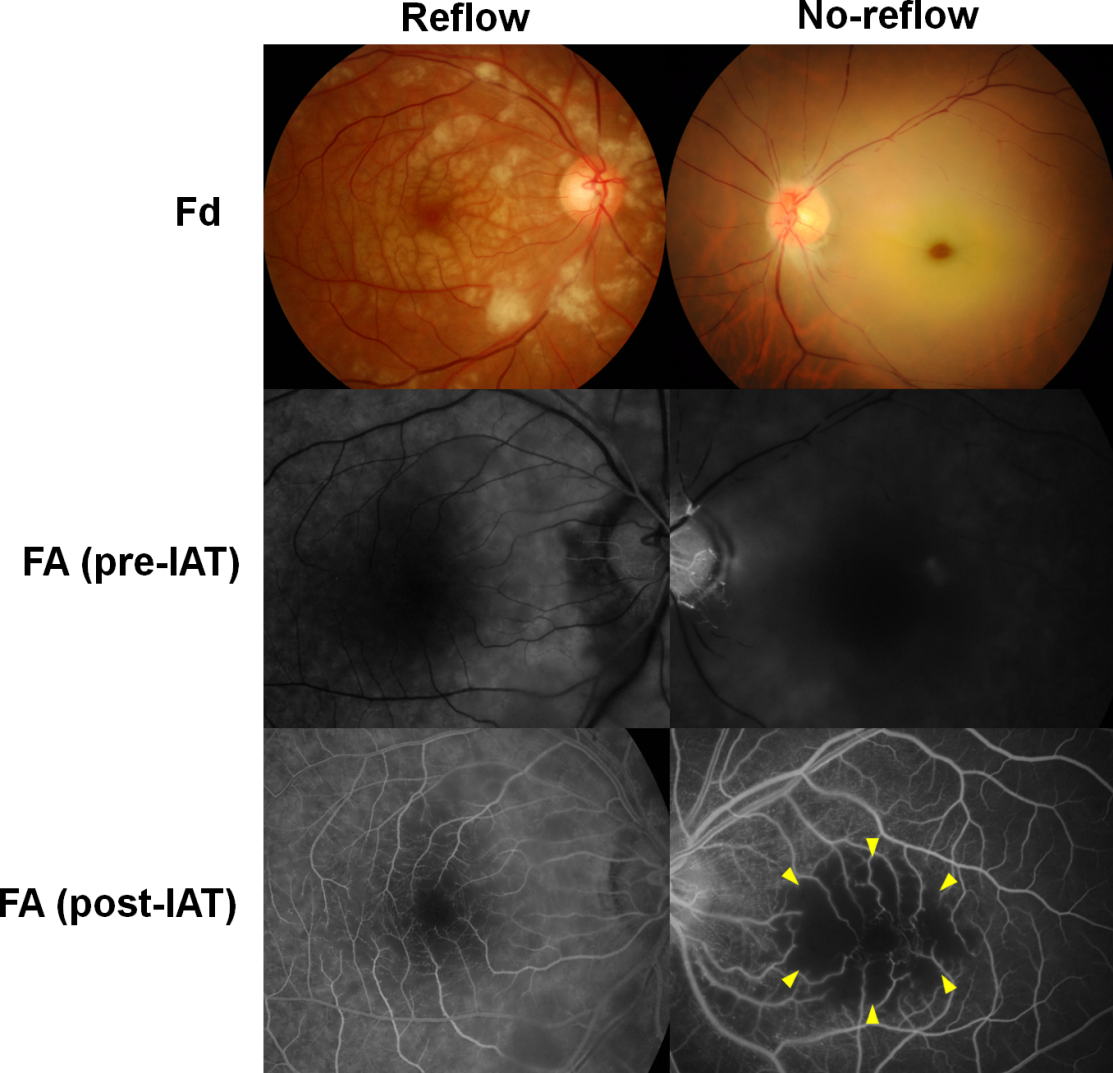
Fundus photographs (Fd) and fluorescein angiography (FA) images of no-reflow (left) and reflow (right) eyes with central retinal artery occlusion. FA images (obtained 30 s after intravenous fluorescein injection) show improved arterial perfusion following intra-arterial thrombolysis (IAT) in both eyes, although a round area (yellow arrowheads) exhibiting capillary nonperfusion (and consequently retinal tissue nonperfusion) in the macula is observed only in the no-reflow eye.

CRAO was defined by delayed central retinal artery filling on FA and ischemic whitening of the retina on funduscopy in patients with sudden vision loss (Fig 1). Permanent CRAO patients who met our eligibility criteria for thrombolysis[16] were considered for IAT, which was performed as previously described[16] after obtaining written informed consent. Other patients with permanent CRAO were treated with conservative treatment, including ocular massage (repeated manual compression for 10–15 s, followed by sudden release, using a 3-mirror contact lens for 3–5 min) and intraocular pressure-lowering agents (topical timolol 0.5%, oral acetazolamide 500 mg). The institutional review board at Seoul National University Bundang hospital approved our study, which adhered to the tenets of the Declaration of Helsinki. Written informed consent for the use of clinical records was not given by patients but the records or patient information was anonymized and de-identified prior to our analysis.

**Fig 2:**
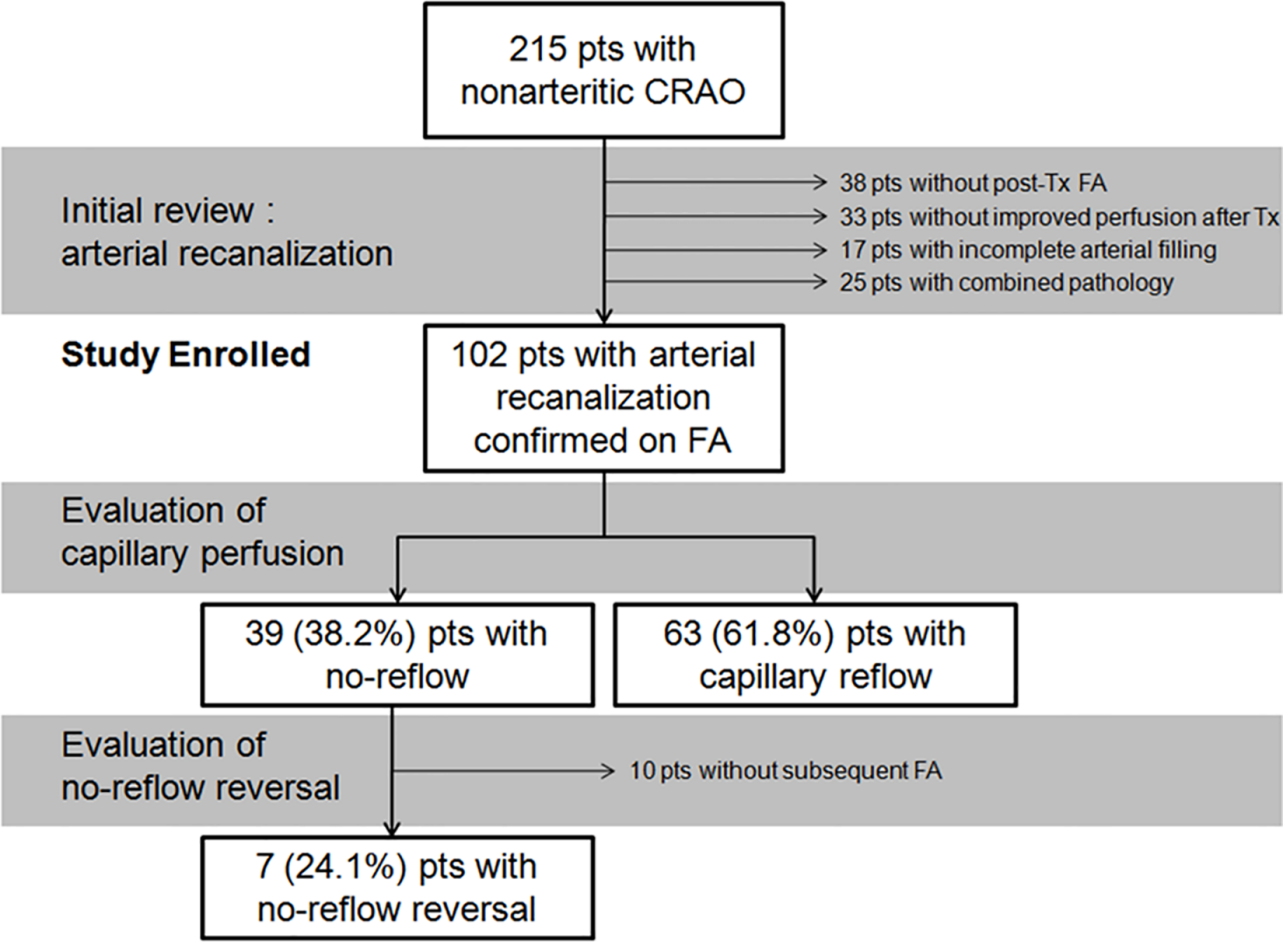
Incidence of the no-reflow phenomenon and its reversal in patients with central retinal artery occlusion (CRAO). The no-reflow phenomenon was defined as the lack of tissue (capillary) reperfusion after successful arterial recanalization in this study. Among the 102 enrolled patients whose arterial recanalization was confirmed by fluorescein angiography (FA) images, 39 showed tissue (capillary) nonperfusion, resulting in a 38.2% incidence of the no-reflow phenomenon. Its reversal could be evaluated in 29 patients, 7 (24.1%) of whom showed reversal of no-reflow.

Fundus FA images and the medical records of 215 consecutive patients diagnosed with non-arteritic CRAO from May 2008 to January 2015 who were followed for ≥2 months were retrospectively reviewed. Those in whom arterial recanalization was obtained either spontaneously (at baseline) or by conservative treatment or IAT were enrolled in this study. Among 196 patients with permanent CRAO treated by conservative treatment or IAT, those without post-treatment FA (N = 38), those with no improvement in arterial occlusion (arm to retina fluorescein appearance time or arm to retina time [17] following treatment was equal to or greater than that at baseline, N = 33), and those with an incomplete arterial phase during angiography (venous phase did not appear on FA, N = 17) were excluded because the evaluation of arterial recanalization was impossible or the tissue non-perfusion may have originated from incomplete retinal arterial filling. The 25 patients with other combined ocular pathologies such as ophthalmic artery occlusion (N = 8), proliferative diabetic retinopathy (N = 9), and central retinal vein occlusion (N = 8) were also excluded. Eventually, 19 patients with spontaneous arterial recanalization on baseline FA (referred to as transient CRAO[14]) and 83 with permanent non-arteritic CRAO showing angiographic evidence of improved retinal artery perfusion following conservative treatment or IAT were included in this study (Fig 2).

### Angiographic Evaluation

Standardized fundus photographs and FA images were acquired at baseline, and FA was additionally performed within 1 week after conservative treatment or IAT. FA images obtained at 1-month and subsequent visits were reviewed for evaluation of no-reflow reversal. According to the baseline funduscopic and angiographic findings, which indicate the arterial obstruction degree,[16, 18] we divided CRAO into incomplete, subtotal, and total CRAO.

In the no-reflow eyes, a round area showing capillary dropout was observed in the macula, while in the reflow eyes, capillary filling was noted in the macula except the foveal avascular zone, which is normally observed on FA images (Fig 1). Accordingly, the no-reflow phenomenon was defined as the presence of a macular area showing capillary dropout or non-reperfusion, excluding the normal foveal avascular zone, in the venous phase of FA (when fluorescein dye passed the retinal arteries and capillaries). Images were analyzed by two independent reviewers (SJA and NKR) blinded to patient data. Positive patients were those in whom the no-reflow phenomenon was identified by both observers.

### Retinal Structural Changes

The structural integrity of the 10 retinal layers identified by OCT was assessed (Spectralis OCT; Heidelberg Engineering Inc, Heidelberg, Germany) and changes in specific retinal layer thickness or reflectivity were compared between the CRAO and contralateral normal eyes. The central macular thickness (CMT) was measured using a circular map analysis protocol, which measures the distance between the first signal from the vitreoretinal interface and the signal from the outer border of the retinal pigment epithelium and calculates the average thickness in a 1-mm diameter circle centered on the fovea. Macular edema (ME) was defined as CMT > 300 μm.[19]

Retinal thickness changes (baseline CMT minus follow-up CMT) were compared between no-reflow and reflow eyes. The frequencies of ME and other morphologic OCT features were compared between groups.

### Statistical Analyses

Kappa statistics (κ) were used for agreement between observers. Baseline demographic/clinical characteristics, CRAO stage, and OCT features were compared between no-reflow and reflow eyes using Student’s t test or the chi-square test to identify associated factors. Multivariate logistic regression using forward selection determined the associated risk factors.

Visual acuity measurements were converted to the logarithm of the minimum angle of resolution (logMAR) for all analyses. The numeric scores for profound low vision (e.g., hand motion, light perception, and no light perception) were substituted for logMAR values as proposed by Lange et al.[20] Final BCVA and CMT were compared between no-reflow and reflow eyes. Multivariate stepwise linear regression analysis identified significant predictors of the final BCVA.

Data for continuous variables are expressed as means ± standard deviations, where applicable. Statistical analyses were performed using SPSS for Windows (Ver. 18.0, Statistical Package for the Social Sciences, SPSS Inc., Chicago, IL). P-values of < .05 were considered statistically significant.

## Results

### Patient Characteristics and No-Reflow Prevalence

Table 1 shows the patients’ clinical and demographic characteristics. The incomplete, subtotal, and total CRAO groups included 32, 63, and 7 eyes, respectively. The mean duration from symptom onset to first visit was 17.9 ± 24.5 h, and the mean follow-up period was 12.3 ± 14.1 months.

**Table 1:** Clinical and demographic characteristics of the study patients.

Characteristic	Number (%) or mean
**Male:Female, patients (%)**	65:37 (63.7%:36.3%)
**Mean age at presentation, years**	61.3 ± 14.1 (range: 22–85)
**Treatment, observation:conservative treatment:IAT (%)**	19:8:75 (18.6:7.8:73.5)
**Mean follow-up period, months**	12.3 ± 14.1 (range: 2–57)
**Time from symptom onset to visit, hours**	17.9 ± 24.5 (range: 1–168)
**Underlying disease/risk factor**	
Diabetes mellitus (%)	22 (21.6%)
Hypertension (%)	57 (55.9%)
Hyperlipidemia (%)	19 (18.6%)
Current smoking (%)	15 (14.7%)
History of stroke (%)	14 (13.7%)
History of ischemic heart diseases (%)	11 (10.8%)
Current use of antiplatelet agent (%)	24 (23.5%)
**CRAO stage, incomplete:subtotal:total (%)**	32:63:7 (31.4%:61.8%:6.9%)
**Baseline best-corrected visual acuity, logMAR**	2.22 ± 0.45 (range: 20/50—NLP)
**Baseline central macular thickness, μm**	401.7 ± 159.9 (range: 213–925)

CRAO = central retinal artery occlusion; IAT = intra-arterial thrombolysis; NLP = no light perception

Continuous data are presented as mean ± standard deviation.

Among the 102 patients (65 men and 37 women; mean age, 61.3 ± 14.1 years), the no-reflow phenomenon was judged to be present in 39 and 42 by two independent reviewers, respectively. Thirty-nine patients were commonly judged as positive, showing good interobserver agreement (κ = 0.939). The incidence of the no-reflow phenomenon among overall patients was 38.2% (39 of 102). The incidence among the patients with IAT-induced and conservative treatment-induced recanalization was 42.7% (32 of 75) and 50% (4 of 8), respectively, and that among those with spontaneous recanalization was 15.8% (3 of 19). Although there was no significant difference in the incidence of the no-reflow phenomenon among the three groups stratified according to the method of arterial recanalization (P = 0.069; Fisher’s exact test), the phenomenon was more frequently noted in non-transient CRAO patients in which arterial recanalization was obtained by treatment.

### Risk Factors

**Fig 3:**
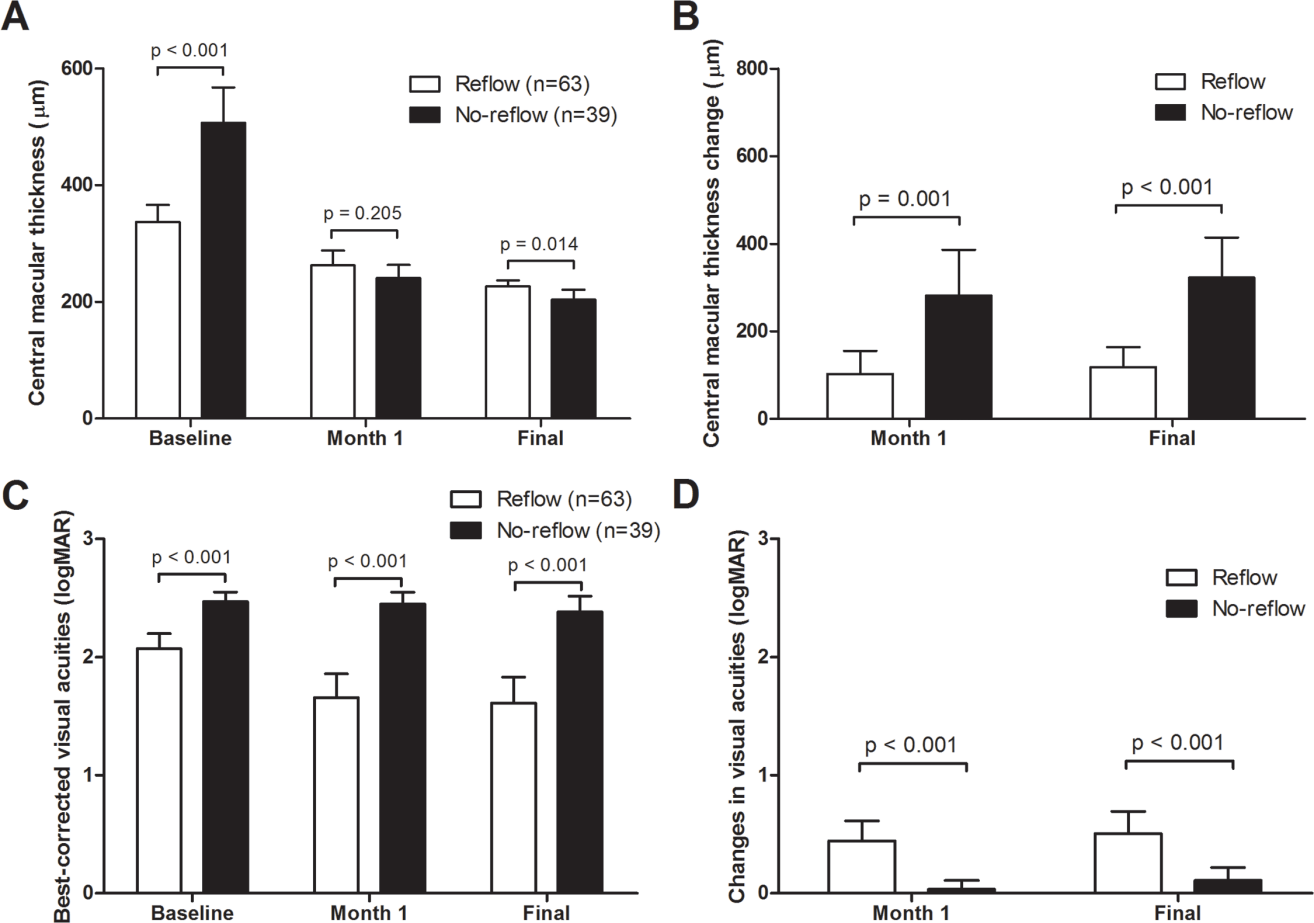
Anatomical and visual outcomes of eyes with and without the no-reflow phenomenon. (A) Temporal patterns of central macular thickness (CMT) and (B) 1-month and final changes in CMT in eyes with central retinal artery occlusion (CRAO). No-reflow eyes show a greater CMT at baseline but a lesser CMT at the final visit compared with reflow eyes. CMT changes at 1-month and final visits are significantly greater in no-reflow eyes. (C) Temporal patterns of best-corrected visual acuities (BCVAs) and (D) 1-month and final changes in BCVA in patients with reflow and no-reflow for CRAO. No-reflow eyes show worse visual function at baseline, 1 month, and the final visit. At the 1-month and final visits, BCVA changes from baseline are greater in the reflow eyes than in the no-reflow eyes. Error bars denote the upper boundary of 95% confidence intervals. *Statistical significance (P < 0.05).

**Fig 4:**
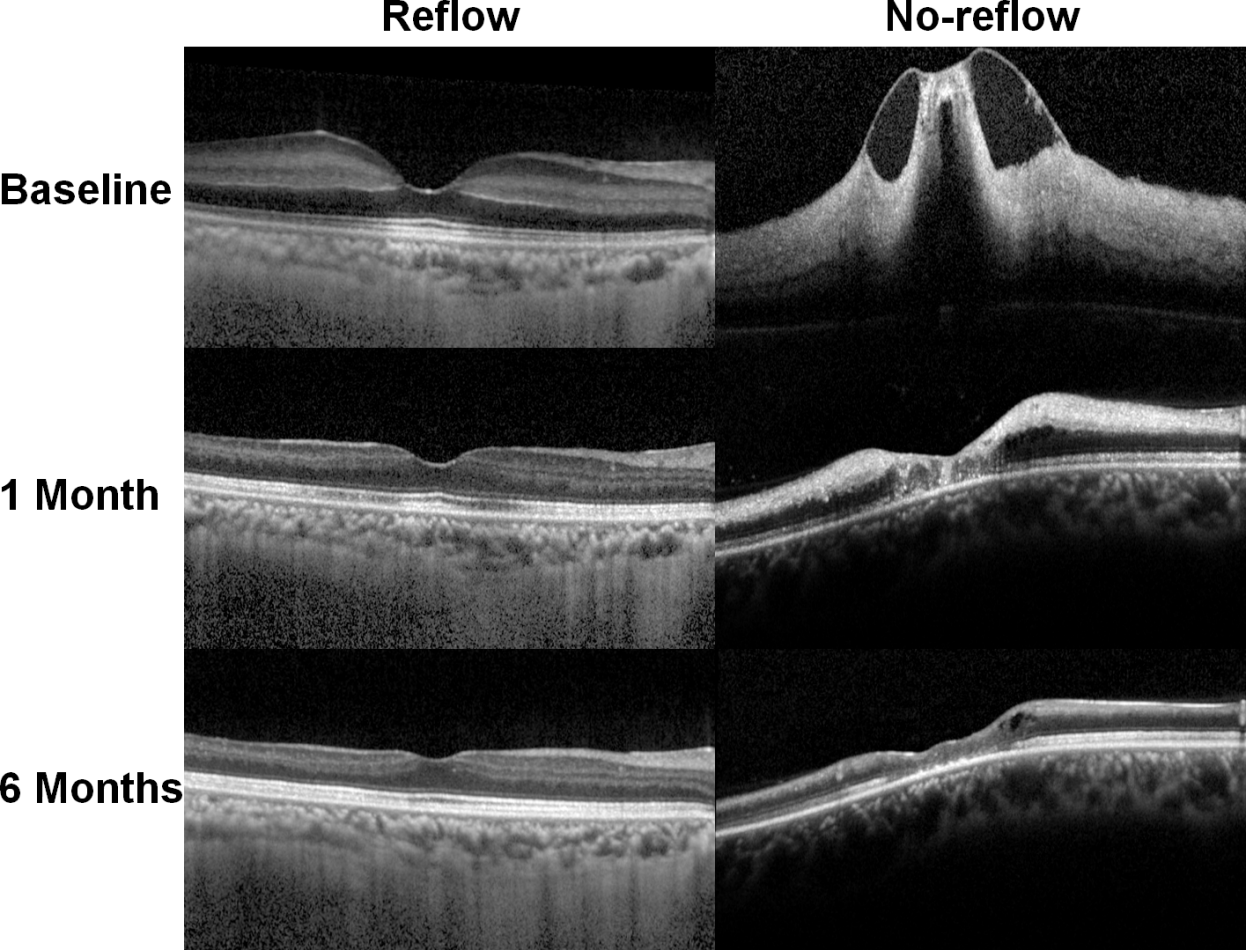
Structural changes in the retina over time in the reflow (left) and no-reflow (right) eyes for central retinal artery occlusion (CRAO). An optical coherence tomography (OCT) image of the no-reflow eye shows loss of layer-by-layer structure in the inner retina and remarkable macular thickening. In contrast, the reflow eye shows a relatively preserved layered retinal structure at baseline (top) and 1 (middle) and 6 months (bottom) later. Furthermore, the no-reflow eye shows more remarkable retinal thinning than the reflow eye and loss of an organized layered retinal structure in the macula.

Table 2 shows the demographic/clinical characteristics, CRAO stage, and OCT features in the no-reflow and reflow eyes. The distribution of CRAO stage was significantly different between groups (P < 0.001; Fisher’s exact test), indicating an association between severe baseline CRAO and the no-reflow phenomenon. Remarkably, only 1 of 32 patients with incomplete CRAO exhibited no-reflow, whereas 33 of 63 (52.4%) with subtotal CRAO and five of seven (71.4%) with total CRAO showed the phenomenon. The baseline CMT was significantly greater in the no-reflow eyes than in the reflow eyes (506.7 ± 173.8 vs. 336.7 ± 109.4 μm, P < 0.001; Student’s t test). The mean baseline BCVA was significantly different between the no-reflow and reflow eyes (2.45 ± 0.23 vs. 2.07 ± 0.49 logMAR, P < 0.001), indicating a significantly more edematous macula and poorer baseline visual function in the former (Fig 3). Among the morphological features identified on OCT (Fig 4), loss of layer-by-layer inner retinal structures and inner retinal thickening was significantly associated with the no-reflow phenomenon, because these eyes showed a significantly higher frequency of these features (94.3% and 100%, respectively) compared with their counterparts (40% and 69.1%, respectively, both P < 0.001).

**Table 2:** Comparison of clinical and retinal structural characteristics between patients with and without no-reflow phenomenon.

Characteristics	Reflow (n = 63)	No-reflow (n = 39)	P value
**Age, years**	59.0 ± 13.8	61.8 ± 12.7	0.411
**Gender, M:F (%)**	32:18 (64:36)	16:10 (61.5:38.5)	0.833
**Risk factors**			
Diabetes mellitus (%)	12 (19.0)	10 (25.6)	0.431
Hypertension (%)	34 (54.0)	23 (59.0)	0.621
Hyperlipidemia (%)	14 (22.2)	5 (12.8)	0.236
Current smoking (%)	10 (15.9)	5 (12.8)	0.672
History of stroke (%)	8 (12.7)	6 (15.4)	0.702
History of ischemic heart disease (%)	7 (11.1)	4 (10.3)	1.0
Current use of antiplatelet agent (%)	19 (30.2)	5 (12.8)	0.045
**Time from symptom onset to visit, hours**	19.2 ± 22.5	15.9 ± 27.4	0.542
**CRAO stage, incomplete:subtotal:total (%)**	31:30:2 (49.2:47.6:3.2)	1:33:5 (2.6:84.6:12.8)	<0.001
**Baseline BCVA, logMAR**	2.07 ± 0.49	2.45 ± 0.23	<0.001
**Baseline CMT, μm**	336.7 ± 109.4	506.7 ± 173.8	<0.001
**Macular edema, No. with CMT >300 μm/OCT performed**	29/55 (52.7%)	31/35 (88.6%)	<0.001
**OCT morphologic features**			
* Findings at Baseline*			
Inner retinal thickening (%)	38/55 (69.1)	35/35 (100)	<0.001
Loss of layer-by-layer inner retinal structure (%)	22/55 (40)	33/35 (94.3)	<0.001
Prominent middle limiting membrane sign (%)	37/55 (67.3)	14/35 (40.0)	0.338
* Findings at the Final Visit*			
Photoreceptor disruption (%)	27/53 (50.9)	26/26 (100)	<0.001

P values were obtained by Student’s t test for continuous variables and by Fisher’s exact test or Chi-square test for dichotomous or ordinary variables.

Logistic regression analyses showed that CRAO stage (P = 0.027) and baseline CMT (P = 0.012) were significantly associated with the no-reflow phenomenon, with an odds ratio (OR) of 4.47 [95% confidence interval (CI), 1.19–16.8] and 1.69 (95% CI, 1.12–2.55) per 100-μm increase, respectively.

### No-Reflow Reversal

**Fig 5:**
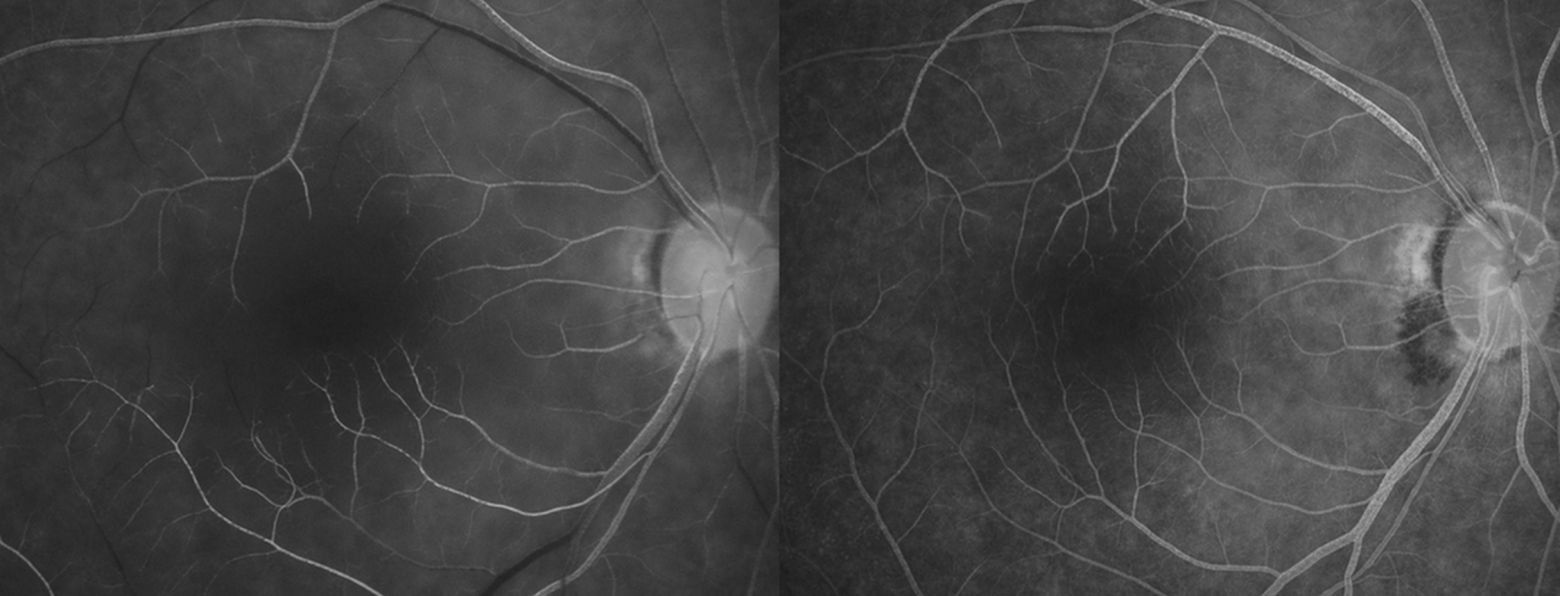
Fluorescein angiography (FA) images of the eye with reversal of no-reflow obtained 3 days (left) and 1 month (right) after treatment (intra-arterial thrombolysis). The macular area in the eye with central retinal artery occlusion shows capillary dropout (nonperfusion) at 3 days (FA image obtained 7 min after intravenous fluorescein injection) and demonstrates reversal of no-reflow at 1 month (FA image obtained 4 min and 30 s after fluorescein injection).

Among the 29 patients with no-reflow who underwent follow-up FA one month later, seven showed capillary reperfusion in the previously nonperfused area (Fig 5); the overall incidence of 1-month no-reflow reversal was 24.1% (Fig 2). All seven patients exhibited capillary reperfusion in the macula at the final visit (mean follow-up: 13.0 ± 9.1 months, range: 2–23 months), whereas the remaining patients exhibited persistently decreased capillary perfusion and remarkable perimacular arteriolar attenuation at subsequent visits, showing a 24.1% incidence of final reversal. CMT [254.3 ± 28.8 and 235.3± 53.7 in eyes with and without reversal, respectively; P = 0.509; Mann–Whitney (M-W) test] and BCVA (2.50 ± 0.24 and 2.44 ± 0.26 logMAR, respectively; P = 0.637; M-W test) at 1 month were comparable between the no-reflow eyes with and without the reversal. The final CMT (227.0 ± 38.8 and 196.3 ± 38.0, respectively; P = 0.113; M-W test) and BCVA (2.34 ± 0.36 and 2.34 ± 0.35 respectively; P = 1.00 by M-W test) were also comparable between the groups, indicating that no-reflow reversal may have no significant impact on the final outcome.

### Visual Outcomes and Retinal Changes

Fig 3 shows the baseline, 1-month, and final BCVA in the no-reflow and reflow eyes. There were significant differences in all BCVA values between groups, indicating a significantly poor visual function at baseline and at subsequent visits in the no-reflow eyes. Fig 3 also shows the comparison of 1-month and final BCVA changes (improvement) from baseline between the no-reflow and reflow eyes. There were significant differences at 1 month and the final visit (both P < 0.001), indicating a significantly greater visual improvement in the reflow eyes. The proportion of eyes with a clinically significant visual improvement (≥0.3 logMAR)[16, 21] was significantly different between groups at 1 month (47.1% vs. 18.8%, respectively; P = 0.009) whereas the difference at the final visit was not statistically significant between the groups (51.0% vs. 38.7%, respectively, P = 0.282). Multivariate stepwise regression analyses showed a significant association between the no-reflow phenomenon and the final BCVA [regression coefficient (B) = 0.370; P = 0.011] and between the baseline and final BCVA (B = 0.862; P < 0.001).

In eyes with CRAO, retinal atrophy was observed on subsequent OCT images (Fig 4). In particular, the inner retina showed thinning with an indiscernible layer-by-layer structure, which was more remarkable in the no-reflow eyes. Among the outer retinal layers, photoreceptor disruption was noted in all no-reflow eyes and 27 reflow eyes (50.9%), showing statistically significant difference between the groups (P < 0.001; Fisher’s exact test).

The final CMT (Fig 3, top left) was significantly lesser (P = 0.014) and the final CMT change (Fig 3, top right) was significantly greater in the no-reflow eyes (P < 0.001) than in the reflow eyes. The final CMT was significantly correlated with the final BCVA (correlation coefficient, −0.364; P = 0.005), explaining the association between visual outcomes and structural changes.

## Discussion

This study demonstrated the no-reflow phenomenon in 38.2% CRAO patients after treatment or spontaneous arterial recanalization. Baseline macular edema and CRAO stage were the most significant risk factors for the phenomenon. This phenomenon was anatomically associated with more severe atrophic changes and retinal photoreceptor disruption and functionally with poorer visual outcomes.

The incidence of the no-reflow phenomenon was 5% to 50% after successful recanalization in patients with myocardial infarction,[5–8] and the incidence in acute CRAO eyes in our study was 38.2%, comparable with that in a recent report on myocardial infarction (35%).[5] Our results suggest that this phenomenon can occur in the brain after intracranial thrombolysis of the culprit artery because the retina is part of the central nervous system. However, visualization of capillary perfusion within the brain and brain tissue changes is very difficult compared with that of retinal capillaries and retinal neurosensory changes. Therefore, retinal imaging, including FA and OCT, in CRAO eyes may be an important tool for investigating the no-reflow phenomenon and may facilitate the understanding of its analogous condition, i.e., terminal branch brain infarction.

In particular, OCT highlighted the differences in retinal structural changes over time between the no-reflow and reflow eyes. Our recent study showed the structure–function relationship in CRAO using SD-OCT.[22] In the report, CRAO eyes with greater retinal atrophy and photoreceptor disruption showed worse visual outcomes.[22] For example, the phenomenon was significantly associated with photoreceptor disruption at the final visit; therefore, the difference in photoreceptor disruption between the no-reflow and reflow eyes may partly explain the significant differences in visual outcomes between groups. Because inner retinal atrophy and associated retinal thinning were also significantly different between the no-reflow and reflow eyes, significant differences in structural changes between groups may have resulted in the differences in visual outcomes.

With regard to the pathogenesis of this phenomenon, capillaries are occluded because of ischemic injury, reperfusion injury, and interstitial and cellular edema that additionally compresses the adjacent capillaries.[4, 5] In this study, the area exhibiting the no-reflow phenomenon corresponded to the area showing edematous changes. Retinal capillaries are present in the inner retina, which shows more remarkable edematous changes than the outer retina on OCT images of CRAO eyes. In addition, the monolayer of retinal capillaries around the fovea may increase the susceptibility of the macular capillaries to the no-reflow phenomenon. However, at subsequent visits, a small proportion of patients (24.1%) in this study showed no-reflow reversal with macular edema resolution, suggesting permanent capillary injury in the other patients. Furthermore, severe ischemia (i.e. subtotal or total CRAO) was associated with the no-reflow phenomenon in our study. In previous studies, electron microscopy showed capillary damage and intraluminal capillary plugging associated with neutrophil infiltration in an animal model of myocardial infarction.[23] Therefore, our data support a multifactorial pathogenesis, including ischemic/reperfusion capillary injury and retinal tissue swelling that impedes blood flow into the retinal capillaries.

Our data also showed that this phenomenon can be found more often in permanent CRAO than in transient CRAO. In the report by Hayreh et al. ^14^, the phenomenon was reported in cases with transient CRAO and there have been no data on the incidence among subtypes of CRAO. We speculate that the no-reflow phenomenon may previously have been neglected in permanent CRAO eyes because these eyes might require little investigation into the cause of vision loss other than persistent retinal nonperfusion. In contrast, FA of transient CRAO eyes may have been thoroughly inspected to identify the cause of vision deterioration; researchers might find that vision loss in some transient CRAO eyes could be associated with the no-reflow phenomenon. Our results show that the no-reflow phenomenon develops more often in more ischemic and longer-lasting CRAO, indicating a pathogenic association between the phenomenon and ischemic damage to the macular capillary endothelium.

The efficacy of IAT is debatable, with previous studies showing variable outcomes.[16, 18, 21, 24] No therapeutic benefits of urgent IAT compared with that of conservative standard treatment for acute CRAO were shown in a recent randomized trial.[21] As shown in our study, the proportion of patients with the no-reflow phenomenon cannot be neglected; consequently, poor visual outcomes in the no-reflow eyes may affect the overall outcomes, resulting in no visual benefits of IAT over the conservative treatment.[16] However, the phenomenon may also explain the stage-dependent efficacy of IAT shown in some studies.[16, 18] Eyes with incomplete CRAO (partial CRAO[25]), which is a milder form of CRAO, were less likely to exhibit the no-reflow phenomenon after IAT in the present study. Therefore, in this subgroup, IAT may be anatomically and functionally beneficial. Furthermore, the recent finding on the association between the degree of ischemia (represented by stage of CRAO) and visual/anatomic outcome[22] can be explained by our finding that more severe stage of CRAO is likely to have the no-reflow phenomenon.

Our patients with CRAO in whom the duration of retinal ischemia is greater than the retinal survival time (4 h in experimental studies[26]), showed considerable recovery of vision in the absence of the no-reflow phenomenon. An experimental study on the retinal survival time in rhesus monkeys showed that the retina suffers irreversible damage within 4 h after arterial occlusion.[26] However, visual recovery can occur in eyes with CRAO lasting for longer than 4 h,[14] and the extreme clamping or cutting of the central retinal artery in those experimental settings[26] may not reflect the actual clinical conditions for human CRAO development. Although Hayreh et al. claimed that visual improvement after the retinal survival time through IAT or any other treatment has no scientific rationale,[14, 15] our results showed structural differences between the no-reflow and reflow eyes. A longer duration of macular nonperfusion in the no-reflow eyes may have resulted in more atrophic changes in the macula, leading to more severe vision loss.

Our findings have clinical implications in the treatment of non-transient CRAO and the prognosis of transient CRAO. Considering that increased CMT and CRAO stage were risk factors for the no-reflow phenomenon in our study, we suggest a modified treatment strategy for CRAO. Patients with less severe CRAO or those without macular edema may undergo treatment for arterial recanalization, because successful retinal artery recanalization may ensure capillary and neurosensory tissue perfusion in these patients, leading to favorable anatomical and functional outcomes. In cases of transient CRAO, presence of the no-reflow phenomenon should be carefully assessed in the macula, as it may lead to greater structural damage in the area and worse visual prognosis, even in patients with a retinal ischemia duration that is shorter than the retinal survival time.

Our study had several limitations. First, the retrospective design may have resulted in intrinsic drawbacks, namely selection bias, although we included all consecutive CRAO patients who visited our hospital. In addition, the variable follow-up periods limited the results, and prospective studies with longer, uniform follow-up periods are needed to draw more definite conclusions regarding the final visual outcomes. However, any possible increase in visual acuity after CRAO usually occurs within the first week after occlusion;[14, 27] therefore, the variable follow-up periods in our study, including short ones in some patients, may not have affected the final visual outcomes significantly.

In conclusion, our study demonstrated that the no-reflow phenomenon can occur in eyes with CRAO. The incidence of 38.2% in our study indicates that it is not rare. CRAO may be a good model for assessing the no-reflow phenomenon, which may lead to worse structural and functional outcomes. Because macular edema and CRAO stage are the most important risk factors for the phenomenon, baseline assessment of macular thickness and determination of the stage may be clinically important for treatment selection and visual prognosis.
